# Valid Consent for Genomic Epidemiology in Developing Countries

**DOI:** 10.1371/journal.pmed.0040095

**Published:** 2007-04-24

**Authors:** Dave A Chokshi, Mahamadou A Thera, Michael Parker, Mahamadou Diakite, Julie Makani, Dominic P Kwiatkowski, Ogobara K Doumbo

## Abstract

Drawing on experience gained from ongoing research in Mali, this paper describes practical ethical challenges relating to the achievement of valid consent in genomic epidemiology.

Genomic research has the potential to improve global health by elucidating basic mechanisms of disease, susceptibility, and resistance, thereby guiding the development of preventive interventions [1]. Recently developed methods for exploring how human genetic variation affects resistance are likely to provide strategic clues about vaccine development for researchers working on malaria, HIV, tuberculosis, and other diseases of the developing world [2].

Valid consent is a process rather than a simple one-off matter of signing a form.

A key scientific challenge facing the genomic epidemiology of common diseases is the vast number of genetic and environmental factors potentially involved. Taken together with the fact that certain genetic effects may be more apparent in some populations than others, this means that genomic epidemiology studies must often include large numbers of participants across multiple populations. Such studies pose not only scientific but also ethical challenges, including how to achieve valid consent for the collection of large numbers of samples and phenotypic information across a variety of diverse, often resource-poor settings.

While there are many statements setting out ethical principles for biomedical research in developing countries [3–5], there are few validated methods by which to apply those principles to large-scale genomic studies. While the concept of “valid consent” as codified in the Declaration of Helsinki has long been established as a cornerstone of ethical research, the interpretation of this concept in practice is not straightforward [6–8] and is further complicated by the fact that what guidance there is on consent tends to be biased towards clinical trials and does not address novel issues raised in genomic studies [8].

Drawing on experience gained from a long-term programme of basic and applied research on malaria in Mali, including smaller-scale genetic studies [9,10], and from current efforts to bring together malaria research groups across multiple centres on four continents, we discuss the practical challenges of defining and obtaining valid consent for genomic epidemiological research in developing countries.

## Ethical Issues Related to Consent

A number of guiding principles for participation in research in developing countries have been established in guidelines, reports, and notes from the field [3,4,11–14]. While these principles provide a helpful framework, they leave unanswered a number of important questions. For example, several guidelines suggest that unfamiliar concepts should be explained using analogies—but provide no guidance for how in practice one might create useful analogies for, for example, concepts such as “gene”, “DNA”, or “genetic database”.

In what follows, and bearing in mind that valid consent is a process rather than a simple one-off matter of signing a form, we consider a number of challenges in relation to the achievement of consent in genomic epidemiology. We do so under five headings: (1) disclosure and comprehension of information, (2) voluntariness, (3) competence, (4) “broad” or “open-ended” consent for future use of samples, and (5) community dimensions of consent [15].

The discussion of these challenges is made more concrete by the accompanying Boxes 1–4, which set out important principles that could be included in an informed consent form. Each principle includes examples of the actual language currently used in such consent forms. In selecting these examples, we drew on existing consent forms used for genetic studies of malaria in Blantyre, Malawi (a collaboration of Malawi College of Medicine, Wellcome Trust Research Laboratories, and Blantyre Malaria Project), at Kilifi, Kenya (a collaboration of Kenya Medical Research Institute and Wellcome Trust Research Unit), Banjul, The Gambia (Medical Research Council Laboratories), Navrongo, Ghana (a collaboration of Navrongo Health Research Centre and Noguchi Memorial Research Institute), and in Bandiagara, Mali (Malaria Research and Training Centre, University of Bamako). These examples show how key elements of the consent form have been addressed in practice. A more detailed exposition of the role of informed consent forms is also included below.

**Box 1. Elements of an Informed Consent Form Addressing Disclosure and Comprehension of Information**

**What is the purpose of research?**

The consent form and information sheet, or their oral equivalents, will include an accessible and easily understandable explanation of the purpose of the research, for example:

*Malaria is caused by a germ that is passed from one person to the other by the bite of a mosquito that carries the malaria germ. It can be particularly severe in children and may cause death. We do not know why some children become severely ill with malaria or why some of those children die from malaria. We think that some children are born with a better ability to resist malaria than others because of their genetic makeup (the way they are because of ancestry). To understand this problem, we need to study children who come to the hospital with severe malaria and compare them to children who have less severe malaria, to other children who are feeling well, and in some cases, to their healthy relatives.*

**What does participation involve?**

The consent form and oral explanation will provide a clear and accessible description of what involvement in the research will mean, for example: What are the duration, procedures, and risks of the study? Will participation involve the taking of blood samples? Will it require home visits or visits to the clinic? Will participants be required to answer questions about their child's health, or that of their relatives? How will complications that arise during research be handled? The distinction between research being conducted and treatment given should be made as clear as possible and the section should affirm that treatment is not contingent upon participation in the research project.

*When a child is admitted to hospital, it is usually necessary to take a blood sample for testing to ascertain the best treatment. This blood sample is not for research purposes but to help in the care of your child. In addition, we are requesting from parents or guardians of all children admitted to the hospital permission to collect a small extra amount of blood for research purposes. (Explain here the amount involved for the child in question—which may depend on their size—e.g., if the quantity is 2 ml the explanation might be “this will be about half a teaspoon-full”.) This sample will be collected at the same time as the blood samples taken for normal investigations. This will not harm your child in any way. If you do not wish to provide this extra blood sample, there are no negative consequences. Your child will receive the best possible treatment either way.*

## 1. Disclosure and Comprehension of Information

To be valid, consent to participate in research must be based on adequate information and understanding. Participants must have sufficient understanding of the purpose of the research, of what participation in the research means for them (and/or their child), of what will happen to samples and records during and after the study, and of what steps are being taken to ensure confidentiality, security, and privacy. In addition, research participants must be aware of the fact that they can withdraw from the research at any time and should know how to go about this. Participants should, in our view, also be informed about the benefits (if any) that are likely to accrue to researchers, individuals, and communities as a result of the research. Each of these elements presents significant difficulty in research settings where linguistic and cultural practices vary considerably. In our experience, three key challenges in this respect are: linguistic and conceptual barriers to conveying key concepts; the design and provision of appropriate education to help participants understand the implications of involvement; and the question of how best to convey the purpose of the research and the value of participation in it.

### Linguistic and conceptual barriers

Linguistic and conceptual barriers are widely discussed in the literature [16]. In genomic research, one of the most intractable challenges is how to convey key genetic concepts in a clear and understandable manner. Experiences from the field show that the process of working out comprehensible language is of crucial importance in conveying such information, whether in writing or verbally [17]. This process will involve researchers, institutional review bodies, funders, and communities jointly working out commonly accepted language, oral and/or written, for particular concepts. It also means translating consent forms and any accompanying information and scripts for oral explanation into local languages and ensuring that they are independently back-translated for validation. If discrepancies in back-translations are found, researchers will need to search for different channels of communication within the community to deal with them. For instance, in genetic studies of malaria in Mali, scientists work with school teachers in the village. If teachers are involved in the building of consent processes, they can continue teaching and responding to questions about the project when the investigators are not present.

### Educational activities

The requirement for comprehensible transmission of information to and discussions about good practice with participants must be tailored to the local setting. In Mali, for instance, researchers collaborate with translators (who are not part of the scientific investigation) in order to convey the content of the consent forms and information sheets in communities with ancestral oral traditions. Having investigators fluent in local languages serves as a safeguard to ensure accurate translation. Messages recorded on audio or video tapes and played during the consent process have also been useful. As a longer-term intervention, researchers can work with linguists in “word creation”. Word creation involves relating a concept like “gene” to attributes of heredity that are already understood in the local language. To increase understanding, complementary creative approaches—such as the use of drawings showing the scale of how much blood is being taken from a child's body—may need to be employed. Comprehension assessment of consent should be used to both validate and refine these methods.

### Explaining the purpose and value of the research

Researchers must aim to develop, possibly using the techniques described above, a clear, concise, and simple way to explain why a study is being performed and how it may add to what is already known. Making clear in what way participation has the potential to contribute to a public good—e.g., the knowledge required to advance development of treatments for disease—is important not just to help explain the purpose of research but also to assist comprehension of why participation may be worthwhile. Community-based genetic studies may learn from vaccine trials: for instance, in safety trials of a malaria vaccine in Mali, researchers worked with a group of adults who subsequently volunteered for a genetic study after the study's aims were discussed with community representatives and broadcast on local radio stations. In this case, the consent process started during the screening period and continued throughout the study with the purpose of delivering information to local administrative, health, and traditional authorities. After every procedure with volunteers, details on study conduct were explained and questions were answered. The most senior staff from the research group visited the study site and spoke with volunteers on a regular basis. At the end of the trial, an assembly—consisting of all volunteers and the full research staff—was held to receive feedback from volunteers on how the study was conducted.

In addition, an official ceremony was organized to present preliminary results (using non-technical language and clear visual aids) and to publicly acknowledge the participation of volunteers as contributors to the ongoing development of a malaria vaccine. It was conducted by the head of the administrative authority in the district, and was attended by international co-investigators, regional and district health authorities, traditional authorities, and all volunteers. It was found that public recognition and the feeling of real partnership contributed to the volunteers' engagement with the study and comprehension of its purposes [14].

## 2. Voluntariness

In addition to being adequately understood and informed, consent must be voluntary—that is, free from coercion [8]. This means that participants must not come under undue pressure to participate while being informed about the potential value of the research.

### Voluntariness and the relationship between research and clinical practice

Consent may be coerced in a number of ways. In resource-poor settings, for example, the provision of health care in research projects may act as an inducement. If participation in research is the only way for participants to gain access to clinical care, or if participants do not adequately understand the difference between research and clinical practice, this may mean that the decision to participate is not voluntary [18]. This can be compounded in genetic association studies of infectious diseases by the fact that blood for DNA samples is often collected during the acute clinical episode, making it almost impossible to clearly separate research from treatment.

Nevertheless, while complete separation of clinical practice from research may be difficult to achieve in such settings, it is vital that researchers and those involved in community engagement activities take all steps they can to minimize inducement effects [19]. This includes making it clear (through repetition and comprehension assessment) to potential subjects that health-care provision is not contingent on participation and ensuring that a similar level of health care is available to non-participating patients. Coercion could also occur as a result of traditional respect for health professionals, leading participants to feel that it would be impolite or that they are not “allowed” to refuse participation. It will be important to bear this possibility in mind in the development of consent processes.

**Box 2. Elements of an Informed Consent Form Addressing Voluntariness**

**How can the participant withdraw from research?**

It will be made clear that the participant has the right to withdraw from research at any time and that this will have no negative consequences, particularly for treatment. This section will include an explanation of how to withdraw and, if applicable, a description of the provisions for withdrawal from future research.

*Taking part in this study is entirely voluntary. You can withdraw your child from the study at any time. Not taking part in the study will not affect current or future medical care for your child in any way. If you decide to withdraw from the study later, please inform any member of staff or make an appointment with the principal investigator of the study.*

## 3. Competence

For consent to be valid it must also be *competently* given. Where research is to be carried out with young children, very elderly people, people with mental health problems, or people with potentially impaired decision-making ability for other reasons (such as distress, influence of medication, or ill-health), researchers must take special precautions to ensure participants' competence [20].

In cases of research on severe illness, the role of researchers and practicing clinicians may become blurred during consent processes. Dividing the consent process into two stages could help ensure that a vulnerable patient's competence is not compromised by their illness or by distress. Clinicians might obtain consent for taking blood when a patient comes in for treatment during an acute clinical episode, but wait until a later stage, when the patient was not under immediate stress, to seek permission to use the blood in research. In remote settings, however, follow-up consent can be difficult to achieve as it may prove difficult to re-contact patients once they have left the hospital setting.

Another issue is obtaining consent from older children and determining the age at which it is appropriate for a child to veto a parent's consent. Rather than specifying an age threshold, it may be more appropriate for the investigator (with review from a local ethics board and following community consultation) to assess on a case-by-case basis the child's maturity and understanding.

## 4. Consent for the Future Use of Genetic Information

Another issue arising in relation to consent is the legitimacy of “broad” consent. Is it acceptable to ask research participants to consent to the long-term storage of their genetic information for use in future, as yet undetermined, research? Such consent might be thought to be neither voluntary nor informed. However, it may be possible to impose limitations to “broad” consent [21]. For example, the scope of possible research could be narrowed to a specific disease, even if the exact nature of all possible future experiments was not clear at that stage. This could be complemented by an ongoing opt-out process such that participants can withdraw from any study at any future point in time, which would require appropriate processes for withdrawal to be developed beforehand.

Even with the “disease-specific” constraint, however, it still could be difficult to communicate the nature of future, often highly technical research projects to participants, particularly in a developing-country context. In addition, even a disease-specific constraint might not be stringent enough to preclude the misuse of genetic data. For instance, genotyping haemoglobin allelic variants—something that could plausibly be done in a project related to malaria—can also reveal whether or not a child is biologically related to his or her parents.

There are other arguments against the disease-specific constraint. The potential importance of clinical and biological interactions between different diseases (e.g., interactions between nutrition, bacterial infection, and parasitic infection) presents a legitimate scientific rationale for expanding the range of use for stored samples beyond a single condition. Understanding general mechanisms of immune regulation (relevant both to bacterial and parasitic infections, for example) key to addressing malaria would be a case in point. Samples could also be useful for studying a vast number of other diseases, regardless of co-infection status.

Despite these problems, it is important to bear in mind the potential for previously collected genomic data to facilitate medical research in unforeseen ways. The potential value of such data is an important and highly significant ethical consideration in its own right. The reuse of samples also allows researchers to collect fewer additional blood samples from very sick children. This would avoid the harms to participants inherent in collecting samples and the time and costs involved in organizing such collections. From an ethical standpoint, narrow approaches to consent have disadvantages as well as advantages.

The challenge is how to prevent or lessen the likelihood of the abuse of genetic data—say for paternity/maternity testing or for research that could result in discrimination against an ethnic group—while still permitting scientists to engage in useful and ethical research. Alternative approaches to the disease-specific constraint on broad consent might be categorized as (1) institutional and (2) permissive.

Institutional approaches to broad consent could rely upon a well-developed ethics infrastructure (including researchers' ethics committees, local Institutional Review Boards, and funders' oversight) to ensure that genetic data will only be used for projects that meet the criteria set out by these overlapping bodies. The International HapMap Project has adopted this approach [22]. If institutional capacity is not believed to be sufficient in a particular locale, one might instead try to follow the permissive approach to consent, which describes all those things that a participant's sample will not be used for, rather than restricting future use to research of a specific disease. From the perspective of protecting sample populations, there are advantages and disadvantages with each of these approaches. The nature of the research, characteristics of the sample populations, and the likelihood that samples would be used for the benefit of sample populations in the future should be used as criteria to determine which approach is settled upon. Identifying and addressing these considerations appropriately will require the development of innovative ways of involving local communities.

**Box 3. Elements of an Informed Consent Form Addressing Future Use of Patient Information**

**What steps are being taken to ensure confidentiality, security, and privacy?**

The participant will be given an explanation of how their confidentiality and privacy will be protected within the study and of what measures are being taken to ensure that data are secure. This will include particulars about who is granted access to personal information and will state that medical information collected will only be for the purposes of research.

*Once we take a blood sample, we assign it a code number. By assigning your sample a number, we separate the name and any other personal information from the sample. Therefore, information about your child's participation in this study will remain confidential. In any reports, participants will be referred to by code number only. Participants' names will not be used in any reports and will not be shared with anyone except the study investigators. All files with information that could identify you will be kept in a secure location, in case we need to talk to you in the future, and will not be released to anyone else.*

**What will happen to samples and records during and after research?**

The form and oral explanation will include information about the time-frame of research and whether genomic information that is being sampled will possibly be used for more than one study. The extent of the participant's consent will also be explained, e.g., whether consent is limited to research on a specified disease or at a single institution, or is permissive outside of specifically prohibited uses. If samples are to be stored after the end of the current study, precise information on how long and where samples will be retained must be included. Forms and explanations should separate the option of consenting to future research projects and consenting to the research project being conducted at that time.

*We will test your child's blood to see how it responds to malaria, and what your child inherited from his/her parents that may have affected his/her ability to respond to malaria. There are other tests to better understand malaria that we are not aware of now that we might wish to perform on your child's samples. With your permission, the blood taken from your child's arm will be kept and stored in our laboratory locally and in the laboratories of our partners abroad who are working with us on this research project. If you allow your child's blood to be stored for research, any future projects using the blood sample would be approved by an ethics review board. However, you may choose not to have your child's blood stored for future research and still be part of this study.*

## 5. Community Dimensions of Consent

In our view, in many developing country settings, consent may be invalid if it does not have a familial or communal dimension. In all cases this will involve some level of community involvement and consultation in the research; in many cases, it will also require appropriate processes of community consent. However, tension may arise between individual and community consent; for example, if community elders decide that research should be participated in but individuals are unwilling, or if community leaders withhold consent but individuals want to participate. Community consent is no substitute for individual informed consent [4], but could be considered a precondition.

For example, in the stepwise approach to consent utilized in malaria trials in Mali, community consent has been seen as the first phase of a continuous process [14,23]. Even before seeking community consent, investigators should identify relevant official and unofficial decision-making structures that exist within communities. If such a multi-step approach is to work, each social unit consulted must have veto power; that is, only if all social units grant consent can it be considered valid.

### The role of consent forms

While it is important to remember that valid consent cannot be attained solely with the completion of a consent form, such forms can play an important role. Among other things, a consent form can help to ensure that consent processes are comprehensive in information conveyed to a research participant. In large-scale genomic research, the consent form has an additional function: that of ensuring that key elements of the consent are standardized across research sites. It has been demonstrated in the United States that depending upon local institutional review to ensure uniformity of consent process in multi-site research is problematic; such difficulties are likely to be much greater in very diverse and resource-poor settings [24]. It is important therefore that such collaborations themselves take on this responsibility. While a standardized consent form is not by itself sufficient to ensure valid consent across research sites, it can contribute usefully to consistent practice.

**Box 4. Elements of an Informed Consent Form Addressing Community Benefit**

**What benefits will accrue to researchers, individuals, and communities as a result of participating in this project?**

A discussion of group benefits from research will be included, as will compensation made available to individual participants. An explanation of the way in which the research is expected to contribute to public knowledge will be included, and, where relevant, a statement of whether the research results will be used commercially.

*Although there are no direct benefits for participating in this study, the knowledge that will be gained will help us develop new ways to control malaria that may benefit people living in areas where malaria is a serious problem. It is hoped that our research will help scientists develop new treatments for malaria, although this may not occur for many years. If this occurs, neither researchers nor participants such as you or your child are expected to receive any financial benefits.*

The development of a consent form for use in a range of diverse sites needs to be flexible enough to take into account local community and study-specific circumstances. The form will also need to address—in a relatively standard format—a number of key issues [25]. These have been broken out in Boxes 1–4 here and include: the purpose of the research, what participation involves, what will happen to samples and records during and after the research, what steps are being taken to ensure confidentiality, security, and privacy, how participants can go about withdrawing from the research, and what benefits will accrue to researchers, individuals, and communities as a result of the project.

## Conclusions

The tools of genomics have great potential for developing sustainable solutions to global health problems. As researchers cover new ground in understanding the fundamental molecular mechanisms of disease, we must make certain that sound ethical practices keep pace with scientific innovation. In this paper we have explored practical ethical issues arising in relation to the achievement of valid, community-informed consent in genomic epidemiological research. The achievement of valid consent for such research takes on particular importance in developing countries, both from the perspective of protecting individuals and building trust between communities and research groups. While funders, research institutions, and international policy makers have an important role in achieving valid consent, we have approached the issue from the perspective of scientists in the field, for it is scientists who ultimately manage the interaction between laboratory and community.

Linking principles to practices at the ground level is the most powerful way to ensure that valid consent is indeed realized. In this paper we have highlighted a number of key areas of practice and policy relating to the achievement and understanding of valid consent in genomic epidemiology in developing countries. In turn, these have suggested a number of areas of practice in which embedded ethicosocial research would be of great value. These include: research into models of community consent and education for genomic epidemiology; the development of models for embedded empirical research on obtaining valid consent; research into the ethical and social factors important in building trust between communities and researchers; and research into the use of broad consent and the secondary research use of data and archived samples.
